# Plain Language Summary

**DOI:** 10.2903/j.efsa.2023.p210501

**Published:** 2023-05-17

## Abstract

This publication is linked to the following EFSA Journal article: http://onlinelibrary.wiley.com/doi/10.2903/j.efsa.2023.7989/full

efsa JOURNAL

11 May, 2023

## Risk assessment of plant protection products on bees

**Disclaimer**

- This plain language summary (PLS) is a simplified communication of EFSA's *Revised guidance on the risk assessment of plant protection products on bees (Apis mellifera, Bombus sp. and solitary bees)*. The full EFSA guidance can be found here.
- The purpose of the PLS is to enhance transparency and inform interested parties on EFSA's work on the topic using simplified language to present a summary of the main findings.

### Overview of Scientific Guidance

- Protecting bees is a high priority for the European Commission (EC), which only approves plant protection products (referred to in this summary as pesticides) if they have no 'unacceptable effects' on the environment.
- In 2013, EFSA issued a guidance document on the risk assessment (RA) of such products on bees (*Apis mellifera*, *Bombus* spp. and solitary bees).
- In 2019, the EC and Member States asked EFSA to review the guidance to take into account new scientific knowledge that has emerged since 2013.
- The revised guidance describes the updated specific protection goals (SPGs), as agreed by risk managers (RMs), that quantify 'unacceptable risks' for RA. The updated RA methodology is aligned with the SPGs.
- The revised guidance is particularly relevant to stakeholders, notably those responsible for assessing and managing the risks of pesticides to bees.

### What was EFSA asked to do/what is not addressed?

- The EC requested EFSA to update the previous guidance and consider the following:
  - previous feedback from stakeholders
  - methods used to assess risks to bees – particularly in relation to new data - new SPGs and the outcome of consultations with stakeholders and Member States
  - latest scientific evidence on the RA of pesticides for bees including data on natural background mortality
  - the list of bee-attractive crops
  - the requirements for higher level testing e.g., field tests
- The guidance relates to Regulation (EC) No 1107/2009 and therefore it does not address the risk from combined exposure to different pesticides that might be used on an agricultural landscape throughout a season
- Although bees might be exposed to different matrices (e.g., extrafloral nectar, honey dew, soil, air), these risks could not be addressed due to lack of data.

### How did EFSA carry out this work?

- EFSA set up a working group to review the 2013 guidance.
- Based on the approach adopted, the working group used different methods, including systematic review of the relevant scientific literature and expert knowledge elicitation (EKE, a structured way to obtain information from experts).
- In parallel with the above developments, stakeholders were consulted and RMs discussed the SPGs.
- Based on the collected information, the outcome of the stakeholder consultation and RMs discussions, the working group defined the best methods for the RA and finalised the guidance.

### What data were used and until when are they valid?

- Scientific literature
- Regulatory files
- Previous opinions (2012 and 2013) and a relevant external report
- Expert knowledge (discussions with the working, hearing experts and EKE)
- There is no expiration date on this guidance, but it may be reviewed in light of emerging evidence.

### What were the outcomes and their implications?

- The guidance provides well-elaborated RA schemes for the main exposure routes of bees to pesticides, via contact and diet.
- These new RA schemes are based on a wider scientific knowledge base than the previous schemes and provide more realistic risk predictions.
- There are more options for exposure refinements and more guidance for higher-tier RA.
- Four specific risk cases are defined:
    - acute-contact risk
    - acute-dietary risk
    - chronic-dietary risk
    - larvae-dietary risk
- The guidance includes a method to assess the risk of metabolites (pesticide breakdown products) and technical mixtures, and discussion on how to reduce risks.
- For the risk to honey bees, the guidance recommends addressing two additional aspects: increasing toxic effects due to long-term exposure to low pesticide doses, and sublethal effects.
- Guidance is also given on identifying when a RA is not needed.
- Although the definition of SPGs is not part of EFSA's remit, support was given to RMs (EC and Member States) who agreed on an SPG for honey bees of 10% as the maximum acceptable colony size reduction following pesticide exposure.
- No such level could be agreed for non-*Apis* bees; the need for rigorous studies to understand the risk of the assessed pesticide use on those bees was agreed.

### What were the limitations/uncertainties?

- Little or no data were found in the following areas:
    - potential contamination of non-treated areas from solid pesticide preparations
    - the behaviour of pesticide residues in different matrices, such as plant tissues
    - the potential exposure level of bees via certain environmental matrices such as extrafloral nectar, honey dew, soil and air
    - biology and ecology for the non-*Apis* genus of bees

### What are the key recommendations for public health authorities, policy makers, industry, research community or others?

The guidance sets out an extensive list of recommendations to improve knowledge beyond the current review:

- Elaborate SPGs for non-*Apis* bees
- Develop additional tests for non-*Apis* bees
- Quantify the link between SPGs and the observed effects in different tests
- Develop validated bee population/ecological models
- Conduct research to allow translation of effects from one bee species to others
- Better describe the growing patterns of crops in different regions/climate conditions of the EU
- Define in detail the agricultural landscape and landscape composition (the relative proportion of habitat types in the landscape) of different regions and growing systems in the EU

### Are there any additional information sources for the reader?

- The susceptibility of bees to harmful effects of pesticides might be increased by other environmental stressors and/or by other pesticides. An EFSA project, ApisRAM, has potential to complement other studies and provide a more realistic RA (https://www.efsa.europa.eu/en/topics/insect-pollinator-health#must-b-project).
- Consult ECHA's website for updates on their ongoing work on the pollinator guidance for biocides
- EC decision on SPGs
